# Skeletal age evaluation using hand X-rays to determine growth problems

**DOI:** 10.7717/peerj-cs.1512

**Published:** 2023-11-22

**Authors:** Muhammad Umer, Ala’ Abdulmajid Eshmawi, Khaled Alnowaiser, Abdullah Mohamed, Huda Alrashidi, Imran Ashraf

**Affiliations:** 1 Department of Computer Science & Information Technology, The Islamia University of Bahawalpur, Bahawalpur, Pakistan; 2 Department of Cybersecurity, College of Computer Science and Engineering, University of Jeddah, Jeddah, Saudi Arabia; 3 Department of Computer Engineering, College of Computer Engineering and Sciences, Prince Sattam Bin Abdulaziz University, Al-Kharj, Saudi Arabia; 4 Research Centre, Future University in Egypt, New Cairo, Egypt; 5 Faculty of Information Technology and Computing, Arab Open University, Ardiya, Kuwait; 6 Information and Communication Engineering, Yeungnam University, Gyeongsan-si, Republic of Korea

**Keywords:** Skeletal age estimation, Bone disorder detection, Machine learning, Data augmentation

## Abstract

A common clinical method for identifying anomalies in bone growth in infants and newborns is skeletal age estimation with X-ray images. Children’s bone abnormalities can result from several conditions including wounds, infections, or tumors. One of the most frequent reasons for bone issues is that most youngsters are affected by the slow displacement of bones caused by pressure applied to the growth plates as youngsters develop. The growth plate can be harmed by a lack of blood supply, separation from other parts of the bone, or slight misalignment. Problems with the growth plate prevent bones from developing, cause joint distortion, and may cause permanent joint injury. A significant discrepancy between the chronological and assessed ages may indicate a growth problem because determining bone age represents the real level of growth. Therefore, skeletal age estimation is performed to look for endocrine disorders, genetic problems, and growth anomalies. To address the bone age assessment challenge, this study uses the Radiological Society of North America’s Pediatric Bone Age Challenge dataset which contains 12,600 radiological images of the left hand of a patient that includes the gender and bone age information. A bone age evaluation system based on the hand skeleton guidelines is proposed in this study for the detection of hand bone maturation. The proposed approach is based on a customized convolutional neural network. For the calculation of the skeletal age, different data augmentation techniques are used; these techniques not only increase the dataset size but also impact the training of the model. The performance of the model is assessed against the Visual Geometry Group (VGG) model. Results demonstrate that the customized convolutional neural network (CNN) model outperforms the VGG model with 97% accuracy.

## Introduction

Skeletal age assessment (SAA) is a method for estimating the real age of a person. The patient’s bone age can also be assessed to see if it is consistent with or exceeds his or her chronological age (CA). If the SAA is accelerated or delayed, there may be an issue with growth. In 2017, the RSNA launched a challenge for the data science community to compute SA more accurately and effectively than current methods (Prevedello et al., 2019). In pediatric radiology, skeletal bone age evaluation is a common technique for assessing bone maturity. Skeletal growth reveals the actual rate of bone growth monitored continuously as the bone expands and then changes in shape and size over time (Iglovikov et al., 2018). Bone age is used to gauge how mature a child’s bones are. The human skeleton changes in size and structure as an individual matures from fetal life to childhood then puberty, and eventually as an adult. For the most part, bone ages match biological ages. While people with slowed bone ages undergo a later development surge, those with advanced bone ages frequently experience an early growth spurt before ceasing to develop (Satoh, 2015).

Despite the fact that no formal medical practice exists, two medical techniques are routinely used: G&P by  Greulich & Pyle (1959) and TannerWhitehouse (TW) by Carty (2002). Seventy-six percent of businesses use the G&P method. It is performed using a reference grid on the complete X-ray image and is used by physicians (owing to its ease and speed). The left hand and the left wrist are preferred over radiographs of the right hand and right wrist for detecting bone age (Wittschieber et al., 2013). Three procedures are used in TW methods: the radial, lunate, and first, third, and fifth fingers’ bones are evaluated, while the seven carpals are examined by the carpal analysis, and the thirteen long or short bones and the seven carpal bones are evaluated by the twenty-bones approach. The TW methodology is a formula used to determine scores. Each bone’s maturity stage is given a step from ‘A’ to ‘I’. The final step is to replace each step with a record before computing the overall score. After then, the full record is changed to skeletal age (Westerberg, 2020). The G&P technique contrasts the patient’s radiograph to the atlas’s closest standard radiograph using a schematic approach. The scoring mechanism used by the TW method is regarded to be more replicable than the G&P method since it is significantly more unbiased than the Atlas method. Contrarily, the TW method required more time to analyze than the G&P method did (King et al., 1994).

The TW method established a set of maturity level indicators that can be used as a quick reference. Large morphological transitions may be detected in hand X-ray imaging as children develop through young life, early age, and maturity. The majority of bones during the early stages of their development are composed of metaphases. One notable aspect of the aging process is the ossification of the pineal body. Even while skeletal age usually corresponds with and is consistent with chronological age, certain people differ greatly from the normal amounts of bone development. Depending on the severity and outcome of the illness, those who are noticeably shorter than average during the growth phase may need special care. Current methods for estimating skeleton age provide a source list of places that conform. The bone age is calculated by the researchers based on similarities between images from source localities in the graphics and the X-ray image. Skeletal bone age analysis is utilized in pediatric radiography for preventative and therapeutic evaluations of endocrine problems (Carty, 2002), children’s growth, and chromosomal anomalies (Poznanski et al., 1978). Due to the distinctive nature of the dominant hand’s bone ossification phases, this procedure is typically undertaken by radiological inspection of the left hand, followed by a comparison to chronological age; a discrepancy between the two concepts denotes issues. Due to their flexibility, low radiation dose, and accessibility of various osteoblast sites, left-hand X-ray images are frequently used for bone growth analysis.

The regions of interest (ROIs) used in TW methods are divided into Epiphysis Carpal (Spampinato et al., 2017). ROI’s growth is broken down into several steps, and each one is given an alphabetical letter that correlates to a mathematical conclusion that varies by growth and sex. The results of all ROIs are added together to get an overall bone age growth. Two procedures are less frequently used than G&P techniques because of the time involved in the analysis, but they provide estimates that are more precise than G&P techniques (King et al., 1994). Their sectional character makes them suitable for computerization. These approaches, like most persons for independent medical evaluation, attempt to replicate a medical approach by extensively depending on input from subject-matter specialists. The major concern in such cases is whether the visual quality specified by field workers or employed in medical procedures is sufficient for developing automated approaches.

Medicinal tomography helps make a range of analytical judgments for bone age. Machine learning (ML) methods have been applied for many years to improve diagnosis in medical image processing. To develop a classifier, original data images are usually paired with labeled images (*e.g.*, tumor positively and negatively, or severe measurement). It is found that a trained model provides a reasonable function from simple input photographs to find true labels. ML algorithms mostly use handcrafted features from the input data. Deep learning (DL) models are preferred for extracting appropriate features from the input data automatically and have high representational capabilities. The ability of the deep neural network in classifying images makes it attractive for researchers in medical image processing (Krizhevsky, Sutskever & Hinton, 2017). Examples of inferential and cognitive tasks involving medical images include detection, categorization, segmentation, and regression. Particularly, the convolutional neural network (CNN) has proven to be quite good at extracting and assessing visual features (Lee & Kim, 2018). The authors applied a CNN-based VGG16 model to detect bone abnormality using a dataset containing X-rays of upper bones (Barhoom et al., 2022).

Children’s bone malformation can result from many conditions that affect individuals, including wounds, infections, or tumors. One of the most frequent causes of bone issues that predominantly affect children is the progressive displacement of bones, which occur due to stresses applied to the growth plates during the development of children. The growth plate can be harmed by a lack of blood supply, detachment from the other part of the bone, or slight misalignment. Problems with the growth plate prevent bones from developing, cause joint distortion, and may cause permanent joint injury. By using hand X-ray pictures to evaluate the skeletal bones, the suggested method will avoid these problems.

SAA plays a crucial role in clinical research that examines children’s physical development. High subjectivity, massive sample error, challenging evaluation processes, and lengthy assessment cycles are only a few of the serious drawbacks of traditional artificial SAA approaches. The major goal of this work is:

• To propose an approach that automatically recognizes characteristics in hand X-ray pictures and applies these characteristics to categorize skeletal bone age to identify growth disorders.
• A simple customized CNN is employed to track the development of the hand’s bones in the bone age evaluation approach.
• The proposed CNN model has shown superior performance when compared with the Visual Geometry Group (VGG) model.
• Lowering the stress on X-ray diagnostic centers to lessen the burden on medical personnel in hospitals and health centers to deliver good services to patients.

The proposed study’s structure is set up as follows: The overview of prior works that is based on the study of bone age assessment methods employing knowledge-based image processing, transfer learning, and machine learning is presented in ‘Related Work’. The description of the dataset, the data preprocessing, the models that were used, and the proposed methodology are all included in ‘Material and Methods’. The performance assessment of the tried methods is shown in ‘Analysis of Results’. Results, along with a discussion. The conclusion is presented in ‘Conclusion’.

## Related Work

Several studies have been published on the development of the automated bone age assessment (BAA) method. These studies can typically be divided into traditional ML and DL methodologies. The TW methodology has been used more frequently than the G&P method due to its modular design, which enables feature extraction from specific regions of interest. To educate the techniques or regressor to estimate skeleton age, the conventional machine learning strategy evaluates many handcrafted properties from specific ROIs. Pal & King (1983) developed the initial method for determining bone age. Many computerized BAA techniques have since emerged.

### Image processing

The majority of conventional automated systems use image processing techniques or manual design using automatic ways to segment the ROIs. Several image preprocessing techniques were used by the researcher (Pietka et al., 2001) to generate ROIs from the tibia and metaphysis and discover any relationships between skeletal bone age and features. After two years, another study combined the bone age evaluation and clinical PACS and offered a new bone age assessment method (Pietka et al., 2003). In 2007, the researcher determined the bone age for each of the four categories of X-ray images based on features of ROIs of seven hand bones. Both studies designed regression models based on automated carpal bone segmentation (Hsieh et al., 2007). The study provided a computerized bone age estimation technique based on geometric features of carpal bones  (Zhang, Tang & Li, 2007). The “BoneXpert” methodology, which draws on both the G&P and TW approaches, was introduced by a study in 2009 (Thodberg et al., 2009).

Giordano designed an automatic bone age assessment system using the TW2 technique in 2010 in Giordano et al. (2010) and Giordano, Kavasidis & Spampinato (2016). A bone age evaluation model using a support vector machine (SVM) classifier was proposed by the researcher (Kashif et al., 2016) after examining the impacts of five image processing approaches for extracting features. In addition, Seok et al. (2016) picked 17 ROIs of the hand bones to construct five groups based on anatomical similarities and designed a bone age evaluation technique in 2016 using the weight of associated groups.

A researcher proposed a bone extraction method that used trigonometric concepts with knowledge of the anatomy of the hand and obtained TW2 staging by combining bone segmentation with Gaussian filtering augmentation (Giordano et al., 2007). Epiphyseal or metaphyseal ROI identification was suggested in the study from which the graphical model retrieved information (Pietka et al., 2001). The digital hand’s atlas was made with 1,400 X-ray images for evenly distributed normal youngsters and produced an automated method for calculating bone age (Gertych et al., 2007). Cao et al. (2000) proposed a method based on the web for estimating bone age that includes an atlas created from a huge database of healthy images from different racial groups.

### Knowledge-based approaches

Decision rules or ML techniques are the main foundations of knowledge-based methodologies. Mahmoodi et al. (1997) showed how to segment the bones of a child’s hand radiological image and gauge the developmental process using decision-theory-based methods. Aja-Fernández et al. (2004) developed a fuzzy technique for transforming the plain language description of the TW3 method into an artificial model for bone age estimation. To mechanically reconstruct 15 bones’ borders and examine the bone age from 13 bones, Thodberg et al. (2009) suggested utilizing BoneXpert. A technique developed by researchers allowed for the automated location of phalanx bones and the derivation of various shapes using segmentation of bones (Mahmoodi et al., 2000).

### Deep learning models

Modern methods for image classification, segmentation, object identification, and many other areas, such as medicine and genomics, have been greatly improved by DL (LeCun, Bengio & Hinton, 2015). DL algorithms have also been utilized for several clinical applications such as the categorization of skin cancer (Andre et al., 2019), detection of diabetic retinopathy from fundus images (Kermany et al., 2018; Gulshan et al., 2016), and the evaluation of lung nodules from CT scans (Setio et al., 2016). DL is a suitable application for skeletal maturity or bone age determination through automated radiograph processing. The age estimation method which involves the comparison of one or more radiological images with a reference standard is being used by researchers for many years. DL methods for bone age determination have already demonstrated extraordinary effectiveness in clinical settings, with accuracy on par with that of human experts (Larson et al., 2018; Kim, 2017; Spampinato et al., 2017; Lee et al., 2017; Mutasa et al., 2018; Wittschieber et al., 2013). State-of-the-art methods like VGG-16 (Simonyan & Zisserman, 2015), Inception-V3 (Szegedy et al., 2016), VGG-19 (Simonyan & Zisserman, 2015), Inception-ResNet-V2 (Szegedy et al., 2017), and Xception (Chollet, 2017), employing deep CNN models were proposed by several researchers.

The DL strategy instead aims to directly encode visual properties (Goodfellow, Bengio & Courville, 2016). Numerous DL techniques have been proposed and tested by the authors for automatically identifying the age of skeletal bones (Spampinato et al., 2017). The results showed that there was an average difference of 9.6 months between the results and the expert interpretation. A method that made use of several DL designs, such as U-Net (Ronneberger, Fischer & Brox, 2015), ResNet-50 (He et al., 2016), and VGG-style NN (Simonyan & Zisserman, 2015), superior to other standard methods (Iglovikov et al., 2018). AlexNet, VGG16, and GoogLeNet as transfer learning models have all been used in the ImageNet Large Scale Visual Recognition Competition and received acknowledgment. In terms of accuracy, VGG16 is the most accurate of the three, whereas AlexNet is the least accurate (Canziani, Alfredo & Culurciello, 2016). A study (Tang, Chan & Chan, 2019) used the RSNA dataset and employed the transfer learning strategy to train their TjNet architecture created using CNNs. The study focuses on calculating the bone age from the carpal areas utilizing features rather than on the characteristics of the finger (De Luca et al., 2016). On the other hand, methods in Tang, Chan & Chan (2019) and Pahuja & Kumar Garg (2018) used an ANN regressor to determine bone age.

Artificial neural networks and SVM regressors are the most often used conventional machine learning techniques to predict bone age (Dallora et al., 2019). The authors employed MRI rather than X-ray images in this study, in contrast to earlier ones (Tang, Chan & Chan, 2019). The study has also investigated three other characteristics: texture data, number of features, and a histogram of gradients. These features were all extracted from the phalanges regions of bones and used with the Random Forest predictor (Simu & Lal, 2018). Spampinato et al. (2017) developed the first automated DL system utilizing BoNet architecture. A simple CNN architecture with five convolutional layers is used in the model. The designed BoNet outperformed other contemporary DL models including OverFeat, GoogLeNet, and OxfordNet. Similar to this, Lee et al. (2017) discovered that GoogLeNet was the most effective over various CNN architectures at determining bone age. Additionally, to enhance the model’s accuracy, they used pre-processing steps as opposed to BoNet, which does not employ any pre-processing steps, to normalize the input images and eliminate extraneous noise. A high number of images are typically required when training a DL model to prevent underfitting and overfitting problems.

Wibisono & Mursanto (2020) developed a comparable ROIs division in support of their claim that getting particular attributes enhances the regressor’s ability to forecast bone age. Then, DenseNet-121, Inception-V3, and InceptionResnet-V2 were used to train each of these areas, and the training data was passed into a Random Forest regressor. Another study developed an automated method that concentrates on the index finger’s X-ray images rather than the entire hand for bone age prediction (Reddy et al., 2020; Manzoor et al., 2021). Despite having a small test dataset, their method was able to determine the age of the bones from just one index finger. CNNs were also used to develop an end-to-end technique for forecasting bone age (Marouf et al., 2020). The authors took advantage of CNNs’ capacity to calculate bone age based on gender information, even though the gender categorization used was less accurate than ideal (79.60 percent).

## Material and methods

The dataset and models are described in detail in this section. The approach for the proposed techniques is presented first. Then, the next sections provide a detailed description of the dataset and its visualization, respectively, while the last section provides a detailed explanation of the approaches, procedures, and assessment criteria that are employed.

### Overview

**Figure 1:**
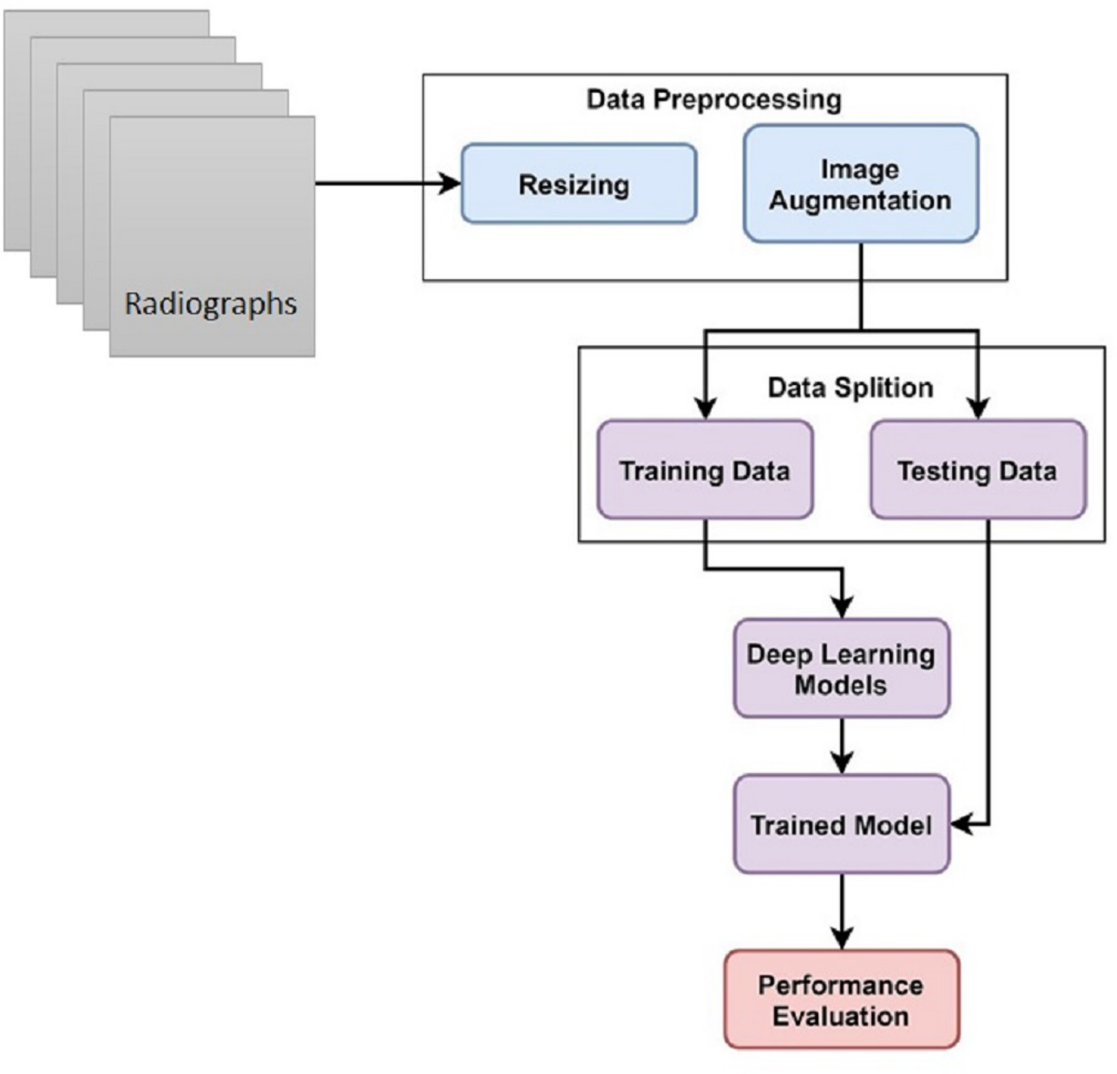
Skeletal age assessment to diagnose growth disorders based on hand X-ray images using deep learning.

This study uses hand X-ray image collection for the diagnosis of growth problems as well as a variety of other ailments, including bone cancer. At first, data is preprocessed by applying image resizing and image augmentation. The data is split into train and test sets in an 80:20 ratio. A customized CNN model is proposed for skeletal growth disorder. The accuracy of the proposed mechanism is then compared to that of the existing approaches. A comparison of the current and suggested approaches is performed to assess the proposed strategy. Figure 1 depicts the proposed methodology for this study.

### Dataset description

An X-ray of a child’s hand was used in a competition to determine the age at the 2017 RSNA. The RSNA Bone Age database (Mader, 2017) is publicly accessible on Kaggle, making it simple to use. This dataset was initially made available as a CloudApp RSNA challenge. The dataset comprises numerous digitally scanned photo files and a CSV with the gender and age of each participant. The datasets used in the Pediatric Bone Age Challenge were contributed by Stanford University, the University of Colorado, and the University of California–Los Angeles. The dataset is comprised of age-related X-ray images of males and females.

#### Data preprocessing

Some preparation techniques, such as data augmentation and image resizing, are used to get good results and develop a strong image classifier. The process of altering an image file’s dimensions is known as picture resizing. Resizing involves increasing or decreasing an image’s size without deleting any of its content. This research work resized all images to 256 × 256 before feeding them to CNN for processing. CNN models require a lot of training data to provide a well-trained model. Image augmentation is mostly used to enhance the performance of the network networks to produce an efficient image categorization with a comparatively modest learning technique. The method of changing already-existing photos to produce more data for the model training procedure is known as image augmentation. This study uses the ImageDataGenerator class for generating more images (Hameed et al., 2021; Umer et al., 2022). Keras provided an image generator class that defines the configuration for image augmentation. Its capabilities include ’ random rotation, shift, shear and flips’, ’whitening’ and ’dimension reordering’, *etc.* Table 1 provides the names and values of the parameters used in the current study.

**Table 1:** ‘ImageDataGenerator’ class hyper-parameters to augment images.

**Paramter**	**Value**
zoom_ range	0.17
rotation_ range	35
height_ shift_ range	0.25
width_ shift_ range	0.25
horizontal_ flip	True
shear_ range	0.20
fill_ mode	nearest

#### Deep learning models

The convolutional neural network has shown robust results in image classification for disease diagnosis (Debbal & Bereksi-Reguig, 2008). This study selected two CNN-based deep neural network models. One is a simple customized CNN model consisting of three convolutional layers. The complete layered architecture along with hyperparameters of customized CNN is presented in Table 2. Other is the transfer learning model namely VGG16 which is a deep and complex model.

**Table 2:** Detail of the hyperparameters used in the customized CNN model.

**Name**	**Description**
Convolution	Filters=(3 × 3, @16), Strides=(1 × 1)
Convolution	Filters=(3 × 3, @128),Strides=(1 × 1)
Max pooling	Pool_ size=(2 × 2),Strides=(2 × 2)
Convolution	Filters=(2 × 2, @256),Strides=(1 × 1)
Average pooling	Pool_ size=(3 × 3), Strides=(1 × 1)
Layer	Flatten()
Fully connected	Dense (120 neurons)
Fully connected	Dense (60 neurons)
Fully connected	Dense (10 neurons)
Sigmoid	Sigmoid (2-class)

CNN is a computationally efficient DL model that uses particular convolution and pooling layers. CNN is the most common type of artificial neural network. A CNN is sometimes linked with a multi-layer perceptron (MLP). Each neuron has an activation function that maps weighted outputs. An MLP becomes a deep MLP when the network adds an additional hidden layer. In a similar vein, CNN is regarded to be an MLP with a specific structure. Due to the architecture of this particular structure, CNN can maintain translation and rotation invariance (Zhao et al., 2017).

The convolutional layer, the first layer in CNN is crucial to convolutional neural networks. The convolution layer seeks to detect the presence of many properties in the input images. Reducing the convolution feature’s dimension is the responsibility of the convolution layer. The feature extraction technique reduces the amount of processing power required to handle huge volumes of data. The models may also be easily learned by extracting rotation and position constant dominating characteristics. A pooling layer’s primary objectives are to reduce the number of variables in a data vector, minimize fitting issues, extract useful information from input convolutions, and limit processing. A non-linear function is known as a rectified linear unit (ReLU), and ReLU(x) = max(0,x). The ReLU correction layer replaces any incorrect input values with 0 to ensure accuracy. It carries out the activation function.

The fully connected layer is a feed-forward neural network. Fully connected layers are the topmost layers of the network. The last fully connected layer of the network classifies the image as input by returning an *N*-dimensional vector, where *N* stands for the size of the class in the image clustering approach. The output of convolution layers is fed into fully connected layers as input.

VGG16 is a well-known and commonly applied classifier model for ImageNet. The winner of the 2014 ImageNet Large Scale Visual Recognition Challenge was the CNN architecture VGG16 (ILSVRC). This one is still regarded as the best visual architecture available today. VGG16 was introduced as a CNN technique by K. Simonyan and A. Zisserman from Oxford University. The concept for the model was presented in 2013, but the final model was not submitted until the ILSVRC ImageNet Challenge in 2014. Due to its ease of use, the VGG16 deep learning picture classification method is well-liked. Given its advantages, VGG16 is commonly used for many applications for vision tasks.

The sixteen-layered VGG architecture trounced the competition in the 2014 ILSVRC, with an error rate of 7.3% and an accuracy of 92.7%. Within a hierarchy of convolution layers (that have varied levels in various styles), there are three fully-connected layers: the first and second have 4,096 connections each, while the third performs 1000-way ILSVRC classification and as a result has 1,000 nodes. The last layer is the softmax layer. For all channels, the fully linked layer configuration is the same.

### Evaluation parameters

Several parameters are used in this study to assess the performance of machine learning models. These assessment measures include accuracy, recall, F1 score, and precision. Using the values from the confusion matrix, we calculate these performance metrics. The four values in the confusion matrix are true positive (TP), false positive (FP), true negative (TN), and false negative (FN).

The percentage of cases that were accurately estimated is known as accuracy. The accuracy score is computed by dividing the number of all predictions by the overall quantity of correct assumptions. The accuracy score has a scale from 0 to 1, with 0 denoting the lowest accuracy and 1 denoting the highest accuracy. The accuracy score is determined as follows

(1) \begin{eqnarray*}Accuracy= \frac{TP+TN}{TP+TN+FP+FN} \end{eqnarray*}

The accuracy of models in use is measured using precision. Precision is computed by dividing the amount of TP by the sum of TP and FP.

(2) \begin{eqnarray*}Precision= \frac{TP}{TP+FP} \end{eqnarray*}

The recall is evaluated by dividing the TP by the total of the TP and FN. The recall score is a numeric value between 0 and 1, with 1 being the greatest possible score.

(3) \begin{eqnarray*}Recall= \frac{TP}{TP+FN} \end{eqnarray*}

The F1 measure sometimes referred to as the F1 score, is a harmonic mean of recall and accuracy. An F1 score is a number between 0 and 1, where 0 is the lowest and 1 is the greatest. Additionally, the F1 score demonstrates accuracy and recall balance.

(4) \begin{eqnarray*}F1score=2\ast \frac{Precsion\ast Recall}{Precision+Recall} \end{eqnarray*}

## Analysis of results

This section summarises the findings from the study and is followed by an in-depth discussion. To calculate the accuracy, deep CNN is trained using the RSNA Bone Age dataset including X-ray scans of the hand.

### Results of customized CNN

Table 3 presents the results of customized CNN. To get the best results, customized CNN is applied using several epochs. Different epochs 10, 20, 30, 40, 50, 60, and 70 were used to analyze results and with 50 epochs CNN attained the maximum accuracy, precision, and F1 score of 97%. Customized CNN, on the other hand, had the highest recall, likewise with 50 epochs, at 96%. Following 97%, the model attained the second-highest accuracy of 96% from 40 epochs and the highest precision of 95% from the same 40 epochs. However, the 95% recall is obtained from 60 epochs and the 95% F1 score is obtained from 70 epochs.

**Table 3:** Performance evaluation of CNN model.

**Epochs**	**Accuracy**	**Precision**	**Recall**	**F1-score**
10	90.0	89.9	90.0	88.0
20	92.1	90.0	89.9	89.9
30	92.4	90.1	90.1	92.0
40	96.0	95.0	94.0	94.0
50	97.0	97.0	96.0	97.0
60	95.0	94.0	95.0	94.0
70	95.0	94.4	94.0	95.0

**Figure 2:**
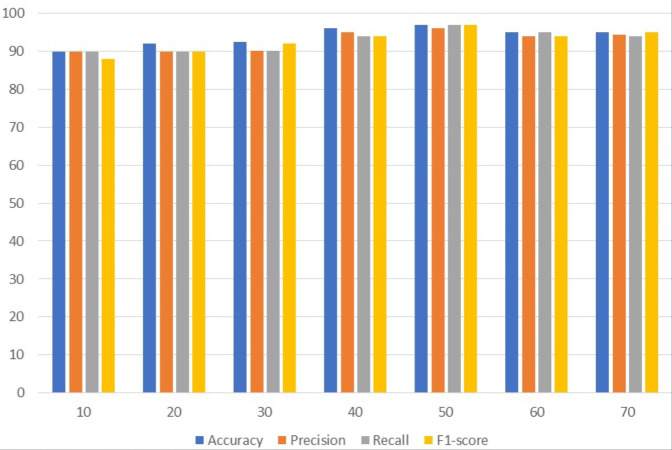
Customized CNN results analyses based on the number of epochs.

Figure 2 uses a bar graph to compare the achieved outcomes for all epochs using customized CNN. From all epochs, customized CNN obtained the best results from 50 epochs. For example, customized CNN obtained 97% accuracy, 97% precision, and 97% F1 score from 50 epochs.

### Experimental results with VGG16

Table 4 contains results using VGG. To get the best results, different epochs are employed with VGG; the best results are obtained using 50 epochs. The VGG model attained the highest accuracy and precision of 96%. With a 97% F1 score, VGG achieved the best F1 score among the other assessment criteria with 50 epochs while simultaneously achieving the highest 95% recall.

**Table 4:** Performance evaluation of VGG model.

**Epochs**	**Accuracy**	**Precision**	**Recall**	**F1-score**
10	88.0	87.0	90.0	89.9
20	91.0	88.9	89.9	90.0
30	93.0	93.0	90.1	90.1
40	94.0	92.0	93.0	95.0
50	96.0	96.0	95.0	97.0
60	93.0	92.0	92.0	91.0
70	90.0	94.4	94.0	94.4

**Figure 3:**
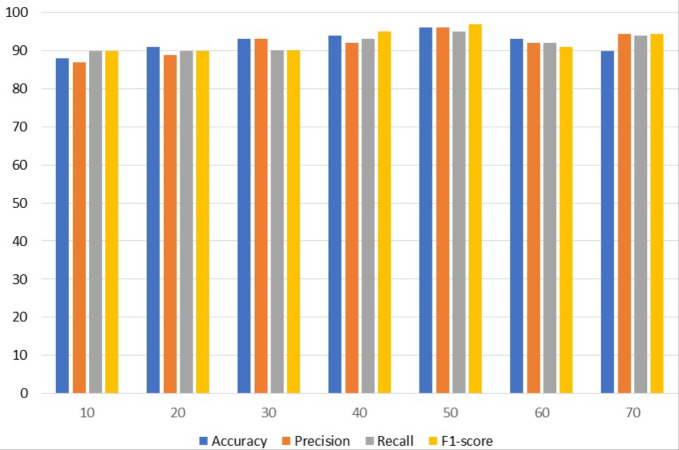
VGG results analyses based on number of epochs.

Figure 3 uses a bar graph to compare the VGG outcomes for all epochs. VGG outperformed all other epochs with results from 50 epochs, including 96% accuracy, 96% precision, 95% recall, and 97% F1-score. This demonstrates that VGG works optimally with a 97% F1 score obtained from 50 epochs.

### Comparison between customized CNN and VGG16

The results of the two models are compared in this section. Table 5 compares the performance of CNN with VGG based on accuracy, precision, recall, and F1 score. Results show that with a 97% accuracy, customized CNN outperforms the VGG model.

**Table 5:** Comparison of the CNN and VGG model performance.

**Epochs**	**Accuracy**		**Precision**		**Recall**		**F1-score**	
	**CNN**	**VGG**	**CNN**	**VGG**	**CNN**	**VGG**	**CNN**	**VGG**
10	90.0	88.0	89.9	87.0	90.0	90.0	88.0	89.9
20	92.1	91.0	90.0	88.9	89.9	89.9	89.9	90.0
30	92.4	93.0	90.1	93.0	90.1	90.1	92.0	90.1
40	96.0	94.0	95.0	92.0	94.0	93.0	94.0	95.0
50	97.0	96.0	97.0	96.0	96.0	95.0	97.0	97.0
60	95.0	93.0	94.0	92.0	95.0	92.0	94.0	91.0
70	95.0	90.0	94.4	94.4	94.0	94.0	95.0	94.4

**Figure 4:**
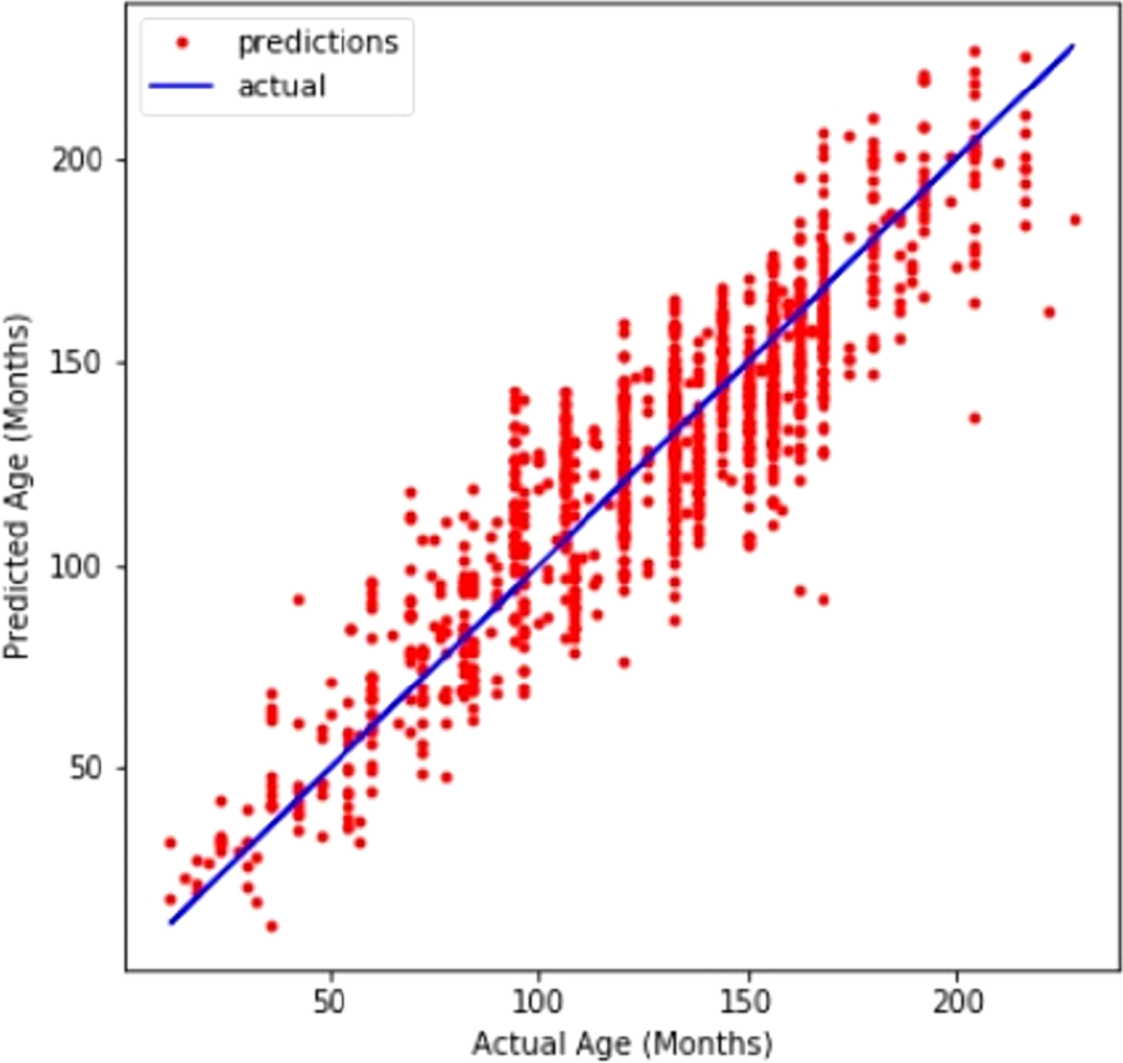
Customized CNN graph between predicted and actual age in months.

A line graph in Fig. 4 compares CNN’s prediction of age with the actual values. The line graph demonstrates that the model performed well.

### Comparison with state-of-the-art approaches from literature

For many years, computer vision and radiological studies have been working towards fully automated bone age assessment. The majority of earlier methods included classification or regression utilizing manually extracted features that were taken from segmented areas of interest (ROIs) for certain bones. Table 6 compares our technique to four previous efforts from the literature. In order to extract visual descriptors and produce fixed-size feature vectors that could be fed into a fully connected neural network, Seok et al. (2012) used a Scale-invariant feature transform and singular value decomposition. Due to the limited amount of images they utilized, their model was not efficient for images that were completely different from their actual dataset. Somkantha, Theera-Umpon & Auephanwiriyakul (2011) determined the carpal bone area utilizing projections in the vertical and horizontal axes. Using the segmented carpal bones, they derived five morphological characteristics and used them in SVM. This technique is similar to the approach of Zhang et al. (Cao et al., 2000) in that hand-engineered features retrieved from carpal bones were utilized as input for a fuzzy logic classifier. The carpal bones are normally fully developed by ages 5 to 7 and do not allow for meaningful distinction after that age, therefore this method is not appropriate for younger children. BoneXpert (Zhang, Gertych & Liu, 2007), the first commercially available and only software-based medical device certified for use in Europe, is the most successful effort to date. In order to autonomously segment bones and to identify the GP or TW2 bone age based on form, intensity, and textural aspects, BoneXpert uses a generative model called the active appearance model. The forecast made by BoneXpert is dependent on the link between chronological and bone ages, therefore it cannot directly determine bone age (Seok et al., 2012). Because of its fragility, the system will not detect radiographs when there is too much noise. According to earlier research, out of 5,161 unique bones, BoneXpert did not detect around 235 of them Thodberg et al. (2008). In spite of the carpal bones’ discriminatory characteristics for young children, BoneXpert does not use them.

**Table 6:** Performance comparison of state-of-the-art models with the proposed model.

**Reference**	**Dataset**	**Method**	**Features**	**Limitations**
Seok et al. (2012)	24 GP female images	scale-invariant feature transform, singular value decomposition Fully connected neural network	SVD-based fixed-sized features vectors from the SIFT description	Lack of robustness to real photos; sparse training and validation data
Somkantha, Theera-Umpon & Auephanwiriyakul (2011)	180 images Cao et al. (2000)	Canny edge detection Fuzzy classification	Morphological features regarding carpal bones	Not applicable to children above the age of seven
Zhang, Gertych & Liu (2007)	205 images Cao et al. (2000)	Canny edge detection Fuzzy classification	Morphological features regarding carpal bones (Capitate Hamate)	Children older than 5 years old for girls and 7 years old for boys are not eligible.
Thodberg et al. (2008)	1,559 images from multiple sources	Active appearance model	Features regarding shapes, intensity, and texture of RUS bones	Prone to excessive noise in photos when input is based on chronological age
Our work	images	Customized CNN	Data-driven, automatically extracted features

In conclusion, the majority of previous attempts at automated bone age estimation have relied on hand-crafted characteristics, which limits the algorithms’ capacity to generalize to the intended application. Our method uses a customized deep CNN to automatically extract key characteristics from all bones on an ROI that was automatically segregated by a detection CNN. Abbreviations used in the whole article are presented in Table 7.

**Table 7:** The acronyms used in this manuscript.

Acronyms	Definition
ANN	Artificial neural network
BAA	Bone age assessment
CA	chronological age
CNN	Convolutional neural network
DL	Deep Learning
FN	False negative
FP	False positive
G & P	Greulich & Pyle
ML	Machine Learning
MLP	Multi-layer perceptron
MRI	Magnetic resonance imaging
ReLU	Rectified linear unit
ROI	Region of interest
RSNA	Radiological Society of North America
SAA	Skeletal age assessment
SVM	Support vector machine
TP	true positive
TN	true negative
TW	TannerWhitehouse
VGG	Visual Geometry Group

## Conclusion

Skeletal age evaluation is a crucial component of the clinical investigation of children’s biological development. Traditional artificial skeletal age assessment techniques have several limitations including high subjectivity, complex models, and protracted assessment cycles. In this work, a deep learning-based automated skeletal bone age evaluation is proposed. By employing a customized convolutional neural network to monitor the development of the hand bones, the proposed skeletal maturity evaluation technique is based on the standards of development of the hand skeleton. Using data augmentation techniques and improving the customized CNN model’s performance, the dataset is enlarged. The final skeletal age evaluation became more accurate as a result of this procedure. Through assessment criteria including accuracy, precision, recall, and F1 score, the performance evaluation of the suggested technique is assessed. Additionally, a comparative analysis is used to assess the proposed approach which proves the robustness of the proposed approach by showing superior performance than a deep and complex transfer learning model. In the future, bone age data normalization and re-scaling of the image pixels will be performed to improve the model’s performance.

## Additional Information and Declarations

### Data Availability

The following information was supplied regarding data availability: The data and code is available at GitHub and Zenodo:
https://github.com/MUmerSabir/PeerJBoneAge. MUmerSabir. (2023). MUmerSabir/PeerJBoneAge: DOI Request (Main). Zenodo. https://doi.org/10.5281/zenodo.7792453.
